# Larval zebrafish maintain elevation with multisensory control of posture and locomotion

**DOI:** 10.1101/2024.01.23.576760

**Published:** 2025-04-21

**Authors:** Samantha N. Davis, Yunlu Zhu, David Schoppik

## Abstract

Fish actively control posture in the pitch axis (nose-up/nose-down) to counter instability and regulate their elevation in the water column. To test the hypothesis that environmental cues shape strategies fish use to control posture, we leveraged a serendipitous finding: larval zebrafish ( *Danio rerio* ) lose swim bladder volume and sink mildly after acute loss of lateral line hair cells. Using long-term (48 h) recordings of unrestrained swimming, we discovered that sinking larvae compensated differently depending on light conditions. In the dark, they swim more frequently with an increased nose-up posture. In contrast, larvae in the light do not swim more frequently, but do climb more often. Finally, after lateral line regeneration, larvae returned to normal buoyancy and swam comparably to control siblings. We conclude that larvae can switch postural control strategies depending on the availability of visual information. Our findings complement and extend morphological and kinematic analyses of locomotion. More broadly, by quantifying the variation in strategies our work speaks to the evolutionary substrate for different balance behaviors.

## Full Text Availability

The license terms selected by the author(s) for this preprint version do not permit archiving in PMC. The full text is available from the preprint server.

