# Supplementary material for: Larval zebrafish maintain elevation with multisensory control of posture and locomotion
Source: bioRxiv. 2025 Aug 2:2024.01.23.576760. Originally published 2024 Jan 24. Preprint. [Version 4] doi: 10.1101/2024.01.23.576760 (PMC10849565; doi:10.1101/2024.01.23.576760)
Supplement: 1 [file NIHPP2024.01.23.576760V4-supplement-1.pdf]

bioRxiv preprint doi: https://doi.org/10.1101/2024.01.23.576760; this version posted August 2, 2025. The copyright holder for this preprint (which was not certified by peer review) is the author/funder, who has granted bioRxiv a license to display the preprint in perpetuity. It is made available under aCC-BY 4.0 International license.

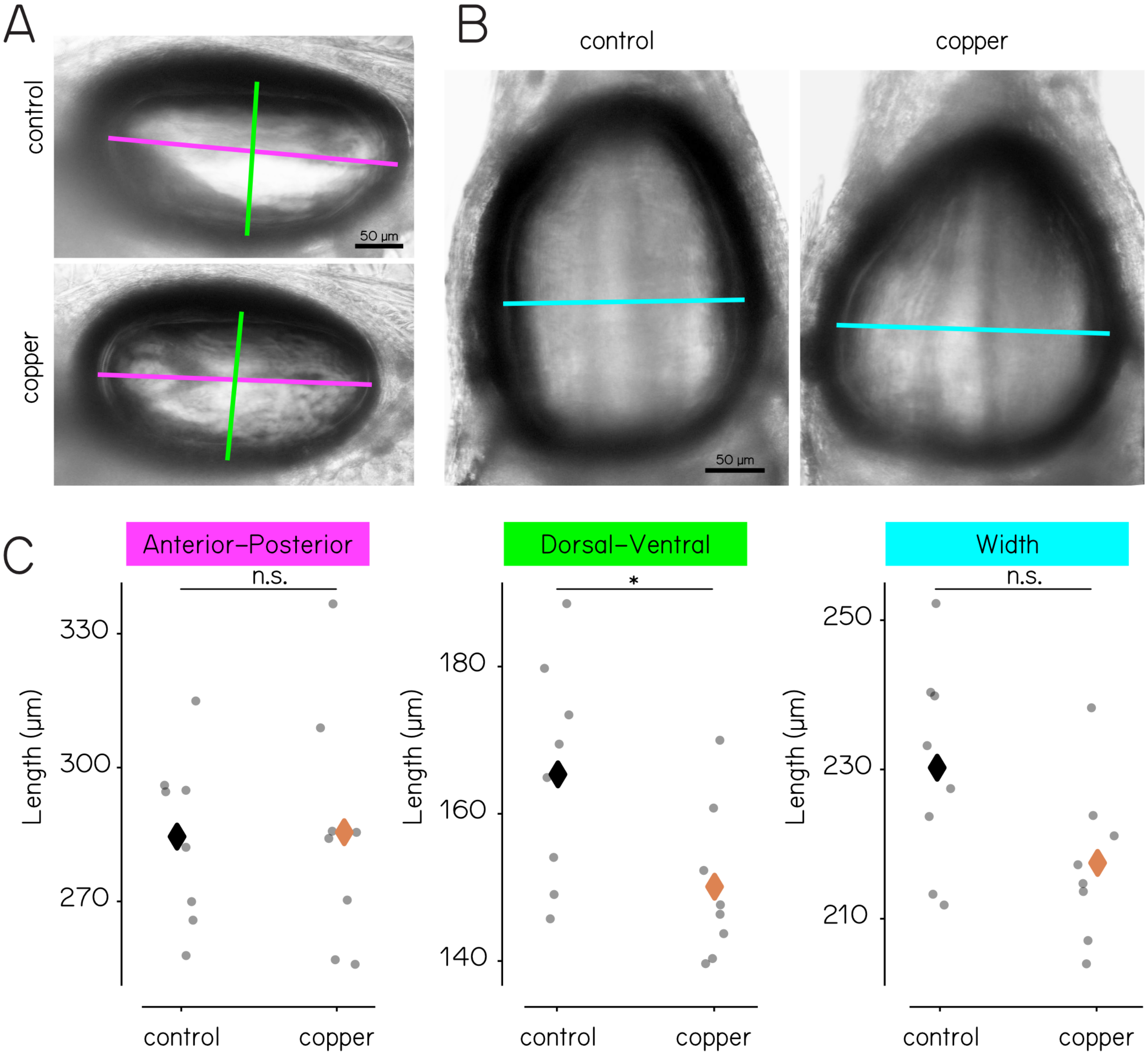

Figure S1: Swim bladder size decreases in the dorsoventral axis after copper treatment.
(A) Example lateral-mount images of swim bladders from a control larva (top) and a different copper-treated larva (bottom) at 7 dpf. Pink axis = anterior-posterior, green axis = dorsoventral.
(B) Example dorsal-mount images of swim bladders from control (left) and copper-treated (right) larvae at 7 dpf. Blue axis = width.
(C) Left: A-P axis length does not change with copper treatment ($p=0.9341$). Middle: D-V axis length decreases with copper treatment ($p=0.0355$). Right: Width does not change with copper treatment ($p=0.0593$). Unpaired t-test.

bioRxiv preprint doi: https://doi.org/10.1101/2024.01.23.576760; this version posted August 2, 2025. The copyright holder for this preprint (which was not certified by peer review) is the author/funder, who has granted bioRxiv a license to display the preprint in perpetuity. It is made available under aCC-BY 4.0 International license.

Table S1: Pre-treatment results for dark-dark and light-dark datasets. Median [interquartile range (IQR)] are listed for non-normal distributions of raw data. P-value calculated using the Mann-Whitney U test ($\alpha = 0.05$), and effect size calculated using Cohen's d. Bold values indicate significance. ˆSteering and righting rotation gain p-value calculated using unpaired t-test (mean and standard deviation listed).

Parameter	Unit	Control	Copper	P-Value [Effect size]
**Dark-Dark Dataset**				
Inter-bout intervals				
Vertical displacement	mm	0.03 [0.59]	-0.02 [0.66]	0.5000 [0.10]
Vertical velocity	mm s^{-1}	-0.04 [0.33]	-0.07 [0.40]	0.4172 [0.10]
Duration	s	1.48 [2.15]	1.52 [2.24]	0.3002 [0.03]
Pitch	deg	14.39 [20.57]	15.81 [18.78]	0.2654 [0.09]
Angular velocity	° s^{-1}	-1.43 [4.23]	-2.09 [4.52]	**0.0473** [0.20]
Bouts				
Peak speed	mm s^{-1}	11.36 [6.14]	11.01 [6.75]	0.1481 [0.07]
Trajectory	deg	20.84 [32.90]	23.37 [30.31]	0.2017 [0.11]
Vertical displ (climb)	mm	0.54 [0.68]	0.56 [0.73]	0.5000 [0.04]
Vertical displ (dive)	mm	-0.17 [0.25]	-0.16 [0.23]	0.2017 [0.09]
Attack angle (climb)	deg	6.37 [6.94]	6.27 [7.66]	0.4172 [0.01]
Attack angle (dive)	deg	-5.45 [5.68]	-5.81 [5.92]	0.1481 [0.08]
Steering rotation gainˆ		0.68 [0.02]	0.66 [0.03]	0.3975 [0.56]
Righting rotation gainˆ		0.17 [0.01]	0.18 [0.01]	0.3027 [0.69]
Fraction of climbs	%	74.58 [0.97]	81.09 [6.77]	0.2017 [0.97]
Fraction of dives	%	24.09 [0.90]	18.14 [7.00]	0.2017 [0.94]
**Light-Dark Dataset**				
Inter-bout intervals				
Vertical displacement	mm	-0.08 [0.19]	-0.07 [0.19]	0.1350 [0.05]
Vertical velocity	mm s^{-1}	-0.15 [0.23]	-0.13 [0.21]	0.2817 [0.11]
Duration	s	0.62 [0.34]	0.65 [0.42]	0.2472 [0.13]
Pitch	deg	0.08 [15.03]	0.62 [15.59]	0.3964 [0.04]
Angular velocity	° s^{-1}	-2.20 [3.85]	-1.97 [3.53]	0.3566 [0.08]
Bouts				
Peak speed	mm s^{-1}	12.41 [7.07]	11.91 [6.53]	0.3182 [0.09]
Trajectory	deg	0.001 [3.31]	0.16 [4.04]	0.4790 [0.03]
Vertical displ (climb)	mm	0.11 [0.19]	0.11 [0.20]	0.4790 [0.01]
Vertical displ (dive)	mm	-0.11 [0.17]	-0.10 [0.25]	0.2474 [0.07]
Attack angle (climb)	deg	1.42 [3.46]	1.28 [3.39]	0.2474 [0.05]
Attack angle (dive)	deg	-0.98 [3.13]	-1.24 [3.20]	0.1860 [0.11]
Steering rotation gainˆ		0.73 [0.03]	0.74 [0.03]	0.5436 [0.31]
Righting rotation gainˆ		0.07 [0.01]	0.07 [0.008]	0.3120 [0.52]
Fraction of climbs	%	48.25 [10.42]	49.18 [12.23]	0.3964 [0.10]
Fraction of dives	%	48.76 [10.13]	47.91 [12.70]	0.3964 [0.09]

bioRxiv preprint doi: https://doi.org/10.1101/2024.01.23.576760; this version posted August 2, 2025. The copyright holder for this preprint (which was not certified by peer review) is the author/funder, who has granted bioRxiv a license to display the preprint in perpetuity. It is made available under aCC-BY 4.0 International license.

Table S2: Effects of lateral line ablation in the dark. Median [IQR] used for non-normal distributions of raw data. N = 114 control larvae, 114 copper-treated larvae. P-value calculated using Mann-Whitney U test (α = 0.05), and effect size calculated using Cohen's d. Bold values indicate significance. ˆSteering and righting rotation gain p-value calculated using unpaired t-test (mean and standard deviation listed).

**Parameter**	**Unit**	**Control**	**Copper**	**P-Value [Effect size]**
Inter-bout Interval				
Vertical displacement	mm	0.02 [0.62]	-0.32 [0.65]	**0.0473** [0.73]
Vertical velocity	mm s^{-1}	-0.01 [0.30]	-0.27 [0.45]	**0.0183** [1.00]
Duration	s	1.78 [2.29]	1.42 [1.15]	**0.0300** [0.29]
Pitch	deg	8.75 [17.74]	12.88 [16.80]	**0.0473** [0.32]
Angular velocity	° s^{-1}	-2.70 [3.71]	-6.59 [7.00]	**0.0060** [1.06]
Bout				
Peak speed	mm s^{-1}	11.48 [6.74]	15.37 [10.36]	0.0718 [0.67]
Trajectory	deg	9.77 [29.33]	13.53 [27.02]	0.1481 [0.17]
Vertical displacement (climb)	mm	0.37 [0.57]	0.53 [0.83]	0.0718 [0.35]
Vertical displacement (dive)	mm	-0.16 [0.23]	-0.21 [0.34]	**0.0473** [0.25]
Attack angle (climb)	deg	3.76 [6.92]	1.14 [6.59]	**0.0300** [0.52]
Attack angle (dive)	deg	-5.25 [5.37]	-6.27 [6.15]	0.1050 [0.25]
Steering rotation gainˆ		0.69 [0.02]	0.74 [0.02]	**0.0367** [1.58]
Righting rotation gainˆ		0.18 [0.01]	0.18 [0.02]	0.6281 [0.31]
Fraction of climbs	%	66 [4]	75 [7]	0.0718 [1.59]
Fraction of dives	%	32 [4]	23 [7]	0.0718 [1.76]

bioRxiv preprint doi: https://doi.org/10.1101/2024.01.23.576760; this version posted August 2, 2025. The copyright holder for this preprint (which was not certified by peer review) is the author/funder, who has granted bioRxiv a license to display the preprint in perpetuity. It is made available under aCC-BY 4.0 International license.

Table S3: Inter-bout interval vertical velocity by time post-treatment. Early and late represent data collected in the first and second 5 hours after the treatment, respectively, for each 24-hour behavior session using Dark-Dark dataset. P-value calculated using Wilcoxon rank sum ($\alpha = 0.05$).

Treatment	Time Post-Treatment	Median [IQR]	P-Value
Control	Early	-0.06 [0.29]	0.14164
	Late	0.02 [0.45]	
Copper	Early	-0.3 [0.20]	0.59081
	Late	-0.29 [0.41]	

bioRxiv preprint doi: https://doi.org/10.1101/2024.01.23.576760; this version posted August 2, 2025. The copyright holder for this preprint (which was not certified by peer review) is the author/funder, who has granted bioRxiv a license to display the preprint in perpetuity. It is made available under aCC-BY 4.0 International license.

Table S4: Number of inter-bout intervals and bouts across datasets. Percentages reflect median fraction and [IQR] of total bouts for that group and time point. P-value calculated using Mann-Whitney U test with α = 0.05 for dark and light datasets, and using one-way ANOVA for longitudinal data. Bold values indicate significance.

Dataset	Condition	IBIs	Climb bouts	Flat bouts	Dive bouts	Total bouts	Climb % P-Value
Dark-Dark	control	31228	22537 (66 [4]%)	681 (2 [0.4]%)	10823 (32 [4]%)	34041	0.0718
	copper	14407	10811 (75 [7]%)	258 (2 [0.5]%)	3630 (23 [7]%)	14699	
Light-Dark	control	174820	81680 (46 [5]%)	5394 (3 [0.4]%)	95460 (51 [5]%)	182534	**0.0013**
	copper	120012	75311 (60 [13]%)	3165 (3 [0.4]%)	48602 (38 [13]%)	127078	
Longitudinal–	control	21526	16150 (69 [12]%)	544 (2 [0.9]%)	7138 (29 [11]%)	23832	0.4717
Early Phase	copperEarly	18504	12205 (68 [8]%)	371 (2 [1]%)	5826 (30 [7]%)	18402	
	copperLate	30536	21072 (62 [9]%)	811 (3 [1]%)	11159 (34%)	33042	
Longitudinal–	control	17020	12149 (71 [7]%)	202 (1 [0.2]%)	4831 (27 [7]%)	17182	0.6314
Late Phase	copperEarly	15902	11270 (68 [2]%)	264 (1 [1]%)	5093 (31 [2]%)	16627	
	copperLate	19892	12242 (71 [20]%)	231 (1 [0.9]%)	5683 (27 [19]%)	18156	

bioRxiv preprint doi: https://doi.org/10.1101/2024.01.23.576760; this version posted August 2, 2025. The copyright holder for this preprint (which was not certified by peer review) is the author/funder, who has granted bioRxiv a license to display the preprint in perpetuity. It is made available under aCC-BY 4.0 International license.

Table S5: Effects of vision on swimming after lateral line loss. Median [IQR] used for non-normal distributions of raw data. N = 150 LD control, 150 LD copper. P-value calculated using two-way ANOVA and Tukey's HSD post-hoc comparison (α = 0.05; bold numbers indicate significance), and effect size calculated using Cohen's d. IBI, inter-bout interval

Parameter	Unit	LD Control	LD Copper	Effect of Light	Effect of Treatment	Interaction Effect
IBI Duration	s	0.69 [0.54]	0.63 [0.50]	**8.4e-13**	**9.6e-3**	**9.5e-3**
Vertical acceleration	mm s^{-2}	-0.04 [0.03]	-0.16 [0.08]	**0.0216**	**1.5e-5**	0.9176
Bout trajectory	deg	-1.29 [18.31]	4.28 [21.82]	**7.4e-8**	**1.17e-3**	0.4744

bioRxiv preprint doi: https://doi.org/10.1101/2024.01.23.576760; this version posted August 2, 2025. The copyright holder for this preprint (which was not certified by peer review) is the author/funder, who has granted bioRxiv a license to display the preprint in perpetuity. It is made available under aCC-BY 4.0 International license.

Table S6: Effects of lateral line loss on swimming in the light. Median [IQR] used for non-normal distributions of raw data. N = 150 LD control, 150 LD copper. P-value calculated using Mann-Whitney U test (α = 0.05; bold numbers indicate significance), and effect size calculated using Cohen's d. ˆSteering and righting rotation gain p-value calculated using unpaired t-test (mean and standard deviation listed).

**Parameter**	**Unit**	**Control**	**Copper**	**P-Value [Effect Size]**
Inter-bout interval				
Vertical displacement	mm	-0.08 [0.20]	-0.09 [0.21]	0.2474[0.08]
Vertical velocity	mm s^{-1}	-0.12 [0.19]	-0.21 [0.28]	**0.0090** [0.52]
Duration	s	0.69 [0.54]	0.63 [0.50]	0.3180 [0.15]
Pitch	deg	-0.37 [14.84]	4.96 [16.71]	**0.0019** [0.46]
Angular velocity	° s^{-1}	-4.07 [4.14]	-4.56 [5.50]	0.1860 [0.13]
Bout				
Peak speed	mm s^{-1}	14.22 [7.66]	13.43 [8.82]	0.2817 [0.13]
Trajectory	deg	-1.29 [18.31]	4.28 [21.82]	**0.0013** [0.38]
Vertical displacement (climb)	mm	0.20 [0.25]	0.21 [0.29]	**0.0415** [0.09]
Vertical displacement (dive)	mm	-0.22 [0.21]	-0.21 [0.24]	0.4790 [0.02]
Attack angle (climb)	deg	1.99 [4.33]	1.71 [4.72]	0.2817 [0.06]
Attack angle (dive)	deg	-2.45 [4.03]	-4.26 [5.09]	**0.0067** [0.41]
Steering rotation gainˆ		0.75 [0.03]	0.72 [0.02]	0.0915 [0.90]
Righting rotation gainˆ		0.09 [0.01]	0.10 [0.01]	**0.2557** [0.59]
Fraction of climbs	%	46 [5]	60 [13]	**0.0013** [1.73]
Fraction of dives	%	51 [5]	38 [13]	**0.0013** [1.65]

Table S7: Effects of hair cell regeneration after lateral line ablation. Median [IQR] used for non-normal distributions of raw data. N=85 control larvae, 94 larvae treated with copper in the early phase (7–8 dpf), and 64 larvae treated with copper in the late phase (14–15 dpf). Data from late phase of recordings. P-value calculated using one-way ANOVA with Tukey's HSD post-hoc comparison (α = 0.05; bold numbers indicate significance) and effect size calculated using Cohen's d and enclosed in []. ^Steering and righting gain p-value calculated using unpaired t-test (mean and standard deviation listed).

Parameter	Unit	Control	Copper Early	Copper Late	One-Way ANOVA P-Value	Control v. Early Post-Hoc	Early v. Late Post-Hoc	Control v. Late Post-Hoc
Inter-bout Interval								
Vertical displacement	mm	-0.05 [0.79]	-0.04 [0.70]	-0.35 [0.89]	**0.0048**	0.9000[0.01]	**0.0122** [0.51]	**0.0095** [0.47]
Vertical velocity	mm s^{-1}	-0.04 [0.31]	-0.03 [0.24]	-0.23 [0.46]	**0.0004**	0.9000 [0.03]	**0.0012** [0.68]	**0.0013** [0.63]
Duration	s	2.50 [3.69]	2.92 [3.49]	1.86 [2.48]	0.1832	0.7858 [0.18]	0.1671 [0.53]	0.4424 [0.32]
Pitch	deg	11.25 [21.51]	7.08 [18.09]	9.57 [19.11]	0.5480	0.5569 [0.28]	0.9000 [0.18]	0.6640 [0.11]
Angular velocity	° s^{-1}	-2.63 [4.77]	-3.02 [4.57]	-5.60 [7.46]	**0.0237**	0.9000 [0.11]	**0.0476** [0.55]	**0.0386** [0.63]
Bout								
Peak speed	mm s^{-1}	10.46 [6.56]	9.88 [5.89]	13.07 [9.91]	**0.0180**	0.3964 [0.13]	**0.0119** [0.55]	**0.0330** [0.43]
Trajectory	deg	17.82 [38.01]	12.52 [35.13]	14.50 [37.83]	0.6417	0.4374 [0.19]	0.2474 [0.07]	0.2474 [0.11]
Vertical displacement (climb)	mm	1.20 [0.98]	1.09 [0.90]	1.52 [1.07]	0.5238	0.3182 [0.15]	0.0067 [0.57]	0.0090 [0.41]
Vertical displacement (dive)	mm	-0.19 [0.39]	-0.14 [0.34]	-0.25 [0.46]	0.2605	0.3964 [0.19]	0.0946 [0.40]	0.0639 [0.21]
Attack angle (climb)	deg	9.77 [6.80]	10.68 [6.21]	7.55 [6.11]	0.0671	0.1860 [0.18]	0.0119 [0.70]	0.0639 [0.46]
Attack angle (dive)	deg	-5.37 [6.00]	-4.33 [6.46]	-5.78 [6.19]	0.3155	0.1860 [0.22]	0.0517 [0.31]	0.4790 [0.09]
Steering rotation gain^		0.67 [0.03]	0.67 [0.06]	0.67 [0.07]	0.9937	0.8980 [0.06]	0.9592 [0.02]	0.9584 [0.02]
Righting rotation gain^		0.16 [0.03]	0.16 [0.02]	0.18 [0.01]	0.4476	0.9026 [0.06]	0.1924 [0.68]	0.3251 [0.50]
Fraction of Climbs	%	71 [7]	68 [2]	71 [20]	0.6314	0.0946 [0.08]	0.2474 [0.08]	0.4790 [0.007]
Fraction of Dives	%	27 [7]	31 [2]	27 [19]	0.6440	0.0781 [0.08]	0.2474 [0.08]	0.4790 [0.007]

bioRxiv preprint doi: https://doi.org/10.1101/2024.01.23.576760; this version posted August 2, 2025. The copyright holder for this preprint (which was not certified by peer review) is the author/funder, who has granted bioRxiv a license to display the preprint in perpetuity. It is made available under aCC-BY 4.0 International license.

bioRxiv preprint doi: https://doi.org/10.1101/2024.01.23.576760; this version posted August 2, 2025. The copyright holder for this preprint (which was not certified by peer review) is the author/funder, who has granted bioRxiv a license to display the preprint in perpetuity. It is made available under aCC-BY 4.0 International license.

SUPPLEMENTAL VIDEO

Video S1

Video S1. Real-time recording of control and copper-treated larvae after treatment. Notably, copper-treated larvae sink between bouts, while controls maintain their position in depth. Related to Figure 2.
